# Optimization and Intra-Laboratory Validation of the Neuro-2a Assay for Tetrodotoxin Detection in Mussels

**DOI:** 10.3390/md24090314

**Published:** 2026-09-08

**Authors:** Alessandra D’Arelli, Silvio Sosa, Sonia Dall’Ara, Monica Cangini, Michela Carlin, Pietro Antonelli, Aurora Dall’Occo, Nicolas Scapin, Giuseppe Arcangeli, Carmen Losasso, Aurelia Tubaro, Marco Pelin

**Affiliations:** 1 Department of Life Sciences, University of Trieste, Via A. Fleming 22, 34127 Trieste, Italyssosa@units.it (S.S.); michela.carlin@units.it (M.C.); tubaro@units.it (A.T.); 2 National Reference Laboratory for Marine Biotoxins, Fondazione Centro Ricerche Marine, V.le A. Vespucci 2, 47042 Cesenatico, Italy; sonia.dallara@centroricerchemarine.it (S.D.); monica.cangini@centroricerchemarine.it (M.C.); 3 Laboratory of Microbial Ecology and Genomics, Istituto Zooprofilattico Sperimentale delle Venezie, Viale dell’Università 10, 35020 Legnaro, Italy; pantonelli@izsvenezie.it (P.A.); adallocco@izsvenezie.it (A.D.); closasso@izsvenezie.it (C.L.); 4 PhD National Programme in One Health Approaches to Infectious Diseases and Life Science Research, Department of Public Health, Experimental and Forensice Medicine, University of Pavia, 27100 Pavia, Italy; 5 National Reference Center for fish, Mollusc and Crustacean Diseaseas, Istituto Zooprofilattico Sperimentale delle Venezie, Viale dell’Università 10, 35020 Legnaro, Italy; nscapin@izsvenezie.it (N.S.); garcangeli@izsvenezie.it (G.A.)

**Keywords:** tetrodotoxin, neuroblastoma cells, food safety, toxicity, detection method

## Abstract

Tetrodotoxin (TTX) is a potent marine neurotoxin responsible for severe seafood poisoning in humans, characterized by neurological symptoms that may be fatal. Originally identified as a natural contaminant of pufferfish (Tetraodontidae family), over the last few years TTX and its analogs have also been detected in other edible marine organisms, including mollusks, gastropods and crustaceans. Consequently, there is a need for rapid, sensitive, and reliable methods for TTX detection in seafood. In this study, a functional assay based on the use of mouse neuroblastoma Neuro-2a cells has been optimized and characterized for TTX detection in mussels. The assay is based on the toxin’s ability to block voltage-gated sodium channels, thereby counteracting the sodium-dependent cytotoxicity induced by veratridine and ouabain. The linear range of the TTX standard curve fell between 0.44 and 33 ng/mL, with limits of TTX detection (LOD) and quantitation (LOQ) of 0.132 ng/mL and 0.439 ng/mL, respectively, and good intra- and inter-day repeatability (RSDr= 15 and 11%, respectively). The assay also detected saxitoxin, which shares the same mechanism of action as TTX, but was less sensitive towards 4,9-anhydro-TTX. The minimum mussel extract dilution of 1:100 did not result in matrix-related interference, allowing accurate TTX quantitation, with a LOQ of 0.54 µg TTX equivalents/kg mussel meat. Given its sensitivity, the optimized Neuro-2a assay represents a promising tool for toxicity-based TTX quantitation in mussels before their consumption.

## 1. Introduction

Tetrodotoxin (TTX) is a potent marine neurotoxin involved in seafood-borne human poisonings characterized by initial gastrointestinal and neurological symptoms progressively evolving into ataxia, loss of coordination, cardiac arrhythmias, seizures, and respiratory failure, potentially leading to death [1,2,3]. The clinical manifestations of TTX poisoning are related to its mechanism of action, namely the high-affinity binding to voltage-gated sodium channels (VGSCs), which physically blocks sodium ion influx in excitable cells, therefore, inhibiting the generation and propagation of action potentials in nervous and muscular tissues [4,5,6].

The exact biosynthetic pathways of TTX are still not fully understood, but a variety of bacterial species, including members of the genera *Vibrio*, *Bacillus*, *Aeromonas*, *Alteromonas*, and *Pseudomonas*, have been identified as major toxin producers [7,8]. The microbial origin of TTX might explain its occurrence across a diverse range of marine organisms, as the toxin, initially in pufferfish (Tetraodontidae family) and once thought to be restricted to this vector, has subsequently been detected in various phylogenetically unrelated marine species, including bivalve mollusks, gastropods, cephalopods, echinoderms, and crustaceans [9,10,11,12,13,14,15]. These TTX-bearing organisms were traditionally associated with subtropical and temperate regions of the Pacific area, but, in recent years, the presence of TTX-containing organisms has increasingly been reported along the European coasts, especially in the Mediterranean Sea [16,17,18,19,20,21,22,23,24,25,26,27,28,29]. In Italy, TTX and its analogues (TTXs) were first reported in Sicilian mussels in 2016 and, since 2017, mainly in bivalves and one echinoderm species from the Adriatic Sea [26,28,30,31,32]. The presence of TTX in edible shellfish along European coastlines has raised concerns for food safety, considering that this toxin is currently not regulated within the European Union (EU) and no maximum permitted level has yet been established in seafood. Only the European Food Safety Authority (EFSA) proposed a safety limit of 44 µg TTXs/kg shellfish meat [3], and the EU legislation prohibits the marketing of fishery products derived from species belonging to the *Tetraodontidae*, *Molidae*, *Diodontidae*, and *Canthigasteridae* families [3,27,33,34,35,36]. To date, the Netherlands is the only EU country to have formally included TTX testing into its official shellfish toxin monitoring program, adopting the EFSA-recommended safety threshold of 44 μg TTXs/kg shellfish meat [22]. In the other Member States, TTX has not yet been included in the list of marine biotoxins officially monitored in live bivalve mollusks, even though TTX levels higher than the EFSA limit have been detected in a few cases. The absence of TTX-tailored monitoring does not ensure the detection of these toxins in mollusks intended for human consumption, but their reliable detection also depends on the availability of rapid and sensitive analytical assays.

So far, several methods for TTX detection and quantitation in edible marine organisms have been developed [8,34,37,38,39,40]. Instrumental and structural analysis techniques, such as LC-MS/MS, immunoassays, aptamer-based assays, as well as biosensors, provide highly specific and accurate information about the toxin profile, but they are expensive, time-consuming and require equipped and experienced laboratories. Moreover, the toxins’ identification and quantitation do not reflect the actual toxicity of a seafood sample, since several toxin congeners with different toxic potencies may be concomitantly present. In contrast, functional assays based on the TTX mechanism of action, such as cell-based assays (CBAs), provide results that are directly related to the potential toxicity of the sample under test [38,39,40,41,42,43]. Among them, the Neuro-2a assay stands out as the most promising cell-based assay for TTX quantification. This method uses Neuro-2a murine neuroblastoma cells to detect toxins targeting the VGSCs, including TTX. This assay, originally perceived to detect saxitoxin (STX), a marine toxin sharing the same mode of action as TTX [44,45], is based on the ability of TTX to inhibit the Na^+^-dependent cytotoxicity induced by the combined action of ouabain (O, an inhibitor of Na^+^/K^+^ ATPase) and veratridine (V, an activator of VGSCs). Cell exposure to these compounds leads to excessive Na^+^ influx and cell death. Thus, TTX prevents Na^+^ entry and, consequently, protects Neuro-2a cells from O- and V-induced cytotoxicity. Cell viability is, therefore, proportional to TTX concentration in the sample [22,44,46]. The Neuro-2a cell-based assay for TTX detection was previously applied also to some TTX analogues, such as 5,11-dideoxy-TTX, 11-norTTX-6(*S*)-ol, 11-deoxy-TTX and 5,6,11-trideoxy-TTX, 4,4a-anhydro-TTX, 11-oxo-TTX, 11-nor-TTX-6,6-diol and its C6-ketone modified derivative with a hydroxy amine compound with maleimide moiety. These TTX analogues were able to counteract the cytotoxic effect of O/V with different potencies and, consequently, they were also detectable by the assay [47,48]. However, although some studies have already employed the Neuro-2a assay for TTX detection in edible marine organisms, no unified and standardized protocol has been established. This is mainly due to the variability of key experimental parameters among laboratories, such as, but not limited to, O/V ratio, cell density, and culture medium composition [22,48,49,50]. In addition, despite the use of the Neuro-2a assay for TTX detection, its analytical performance has been only partly characterized. Limited attention has been devoted to the evaluation of matrix effect, which is essential for the accurate quantitation of the toxin in seafood and for the reliable determination of the assay sensitivity. Over the years, some modifications of the assay protocol have been proposed to improve its sensitivity and specificity, with adjustments to assess the occurrence of toxins activating VGSCs in the samples under analysis [51].

Therefore, the aim of the present study was the optimization and further intra-laboratory validation of the Neuro-2a assay for TTX quantitation, providing a revised standard protocol suitable for TTX quantitation in mussels.

## 2. Results and Discussion

### 2.1. Optimization of the Neuro-2a Assay

**Figure 1:**
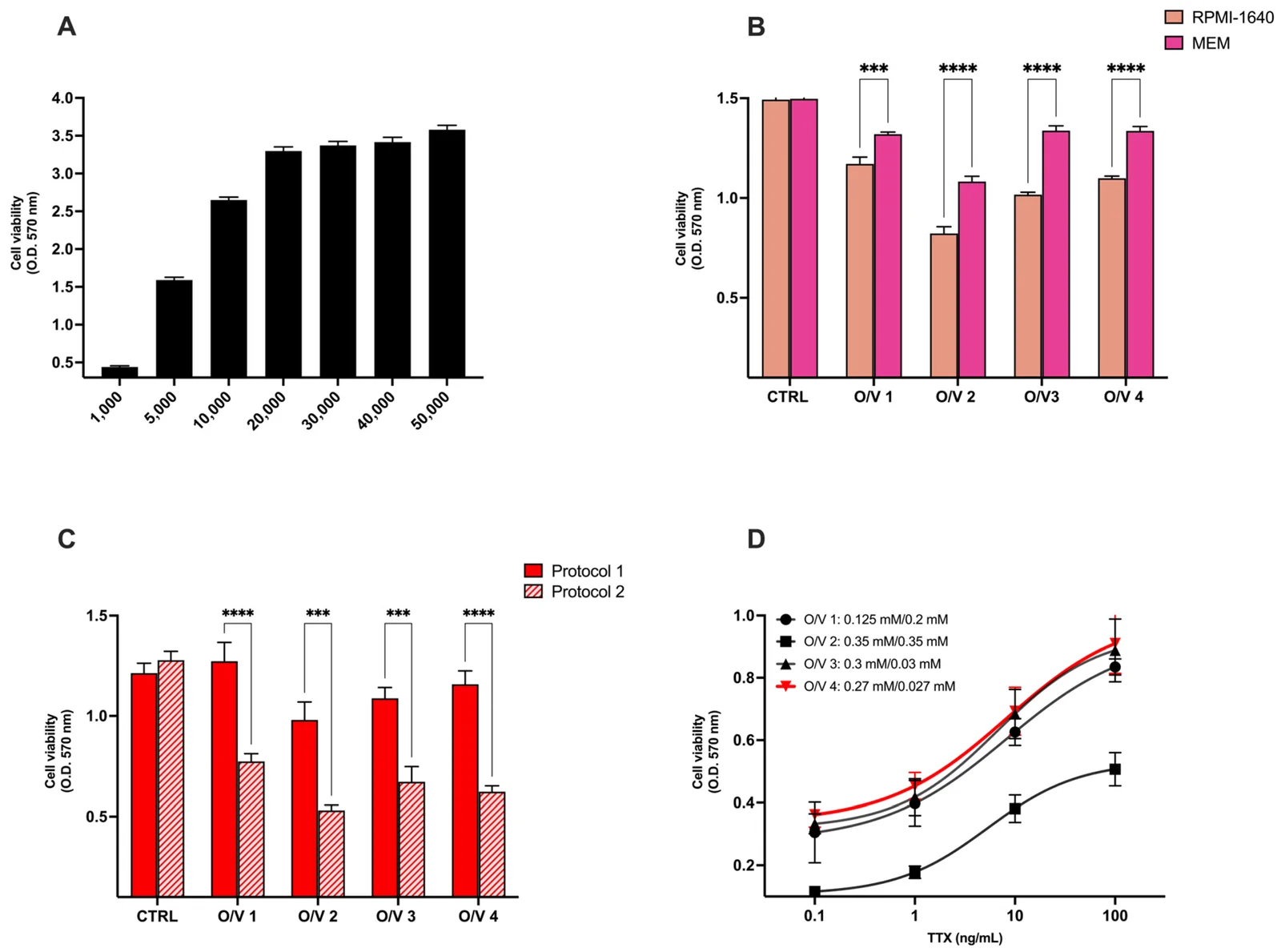
Assessment of the selected parameters to optimize the Neuro-2a assay for TTX detection. (**A**) Assessment of cell density to optimize the reading (O.D.): cells were seeded at densities ranging from 1000 to 50,000 cells/well and cultured for 24 h in RPMI-1640 before cell viability assessment by the MTT assay. (**B**) Assessment of cell media and ouabain (O)/veratridine (V) concentration ratios: 5000 cells/well were seeded and cultured in complemented MEM or RPMI-1640 for 24 h before exposure with each of four ouabain/veratridine (O/V) ratios (O/V 1 = 0.125/0.2, O/V 2 = 0.35/0.35, O/V 3 = 0.3/0.03, and O/V 4 = 0.27/0.027 mM) for further 24 h and cell viability assessment by the MTT assay. (**C**) Assessment of MTT assay procedure: 5000 seeded cells/well were cultured in RPMI-1640 for 24 h and exposed to each of four O/V combinations; cell viability was assessed by the MTT assay following two procedures: (1) direct addition of MTT to the initial culture medium after 20 h exposure to O/V and further cell incubation for 4 h; (2) replacement of O/V containing medium after 24 h of O/V treatment with fresh MTT-containing medium free of O/V and cell incubation for further 4 h. (**D**) Assessment of O/V concentrations ratios in presence of scalar TTX concentrations (0.1–100 ng/mL) for 24 h: 5000 seeded cells/well were cultured in RPMI-1640 medium for 24 h, and exposed to TTX in presence of each O/V combination (O/V 1 = 0.125/0.2 mM; O/V 2 = 0.35/0.35 mM; O/V 3 = 0.3/0.03 mM; O/V 4 = 0.27/0.027 mM). Then, culture medium was replaced with fresh MTT-containing and O/V-free medium and cell viability was measured after 4 h of incubation. Results are reported as optical density values (O.D.) and are the mean ± SEM of at least three independent experiments performed in triplicate. Statistical differences between O/V ratios: ***, *p* < 0.001; ****, *p* < 0.0001 (two-way ANOVA and Bonferroni’s post test).

The cell-based Neuro-2a assay for TTX detection was optimized following the main steps of the original assay for STX detection set up by Manger et al. (1993) [44]. Considering the different variants of the protocol so far reported for TTX detection [22,48,49,50,51], the assay was optimized on the basis of the following undefined experimental parameters that could potentially impact the assay performance (Figure 1): (*i*) density of seeded cells; (*ii*) type of culture medium; (*iii*) MTT assay protocol and (*iv*) optimal O/V concentration ratio.

A suitable seeded cell density was identified to measure an optical density signal at the MTT assay within the upper linear range of the assay, close to the onset of the plateau, to avoid a non-linear increase in optical density. To this aim, Neuro-2a cells were seeded at seven cell densities (1000–50,000 cells/well) in RPMI-1640 and, after 24 h of culture, cell viability was assessed by the MTT assay. At cell densities above 5000 cells/well, the optical density values were higher than 1.5, a threshold above which the readings are no longer linearly proportional to cell number and, consequently, to the amount of formazan produced by viable cells. Therefore, cell seeding densities > 5000 cells/well were excluded from the further steps of the assay optimization. Similarly, a cell seeding density of 1000 cells/well was excluded because the optical density values were close to background signal, thereby compromising measurement reliability (Figure 1A). So, based on these results, subsequent experiments were conducted using a cell seeding density of 5000 cells/well.

As previously mentioned, the Neuro-2a assay enables the indirect detection of TTX through its ability to block VGSCs, thereby preventing the cytotoxic effect induced by the combined action of O and V. Therefore, the subsequent experiments aimed at determining the most suitable O and V concentration ratio. Four different combinations of O and V concentrations and their ratios (O/V 1–OV 4) were selected on the basis of literature data and used to optimize the Neur-2a assay: O/V 1 = 0.125/0.2 mM [48]; O/V 2 = 0.35/0.35 mM [50]; O/V 3 = 0.3/0.03 mM [22,48,49,50]; O/V 4 = 0.27/0.027 mM [22,48,49,50,51]. Moreover, experiments were also carried out to identify the culture medium that provides optimal conditions for cell growth and assay performance. Two culture media were used: MEM, as recommended in the Neuro-2a cell datasheet by the manufacturer (ATTC, Manassas, VA, USA), and RPMI-1640, the medium most used for this assay in the literature. Thus, Neuro-2a cells (5000 cells/well) were seeded in 180 µL of either complemented RPMI-1640 or complemented MEM and cultured for 24 h. Then, cells were incubated with each of the four O/V combinations for a further 24 h (corresponding control cells were incubated with each culture medium alone), and cell viability was assessed by the MTT assay. The viability of control cells did not differ significantly between the two culture media. However, the cytotoxicity induced by the four O/V combinations was significantly higher in cells cultured in RPMI-1640 medium than in those cultured in MEM, indicating enhanced assay responsiveness under RPMI-1640 culture conditions (Figure 1B). Thus, RPMI-1640 was chosen as the optimal culture medium for the Neuro-2a assay.

The next parameter optimized was the MTT cell viability assay, a fundamental step of the original Neuro-2a assay [44]. This widely used assay is based on the ability of mitochondrial dehydrogenases in metabolically active cells to reduce the tetrazolium salt MTT to insoluble purple formazan crystals, quantified spectrophotometrically after solubilization [52]. Two MTT protocols were compared: (*1*) direct addition of MTT to the culture medium 20 h after cell exposure to O/V and further cell incubation for 4 h for a total of 24 h O/V exposure; (*2*) after cell exposure to O/V for 24 h, culture medium replacement with fresh MTT-containing medium and incubation for an additional 4 h without O/V. Significant differences in cell viability were observed when assessed using the two protocols. In particular, when the culture medium was fully renewed with fresh medium containing MTT after O/V treatment (protocol *2*), the viability of cells exposed to each O/V combination was significantly lower than that recorded after direct MTT addition to the original medium in the last 4 h of O/V treatment (protocol *1*; Figure 1C). Based on these results, protocol *2* was selected for further experiments, as it would allow a more evident and stable cytotoxicity induced by O/V.

Lastly, the ability of TTX (24 h exposure to scalar concentrations within the range of 0.1–100 ng/mL, dilution factor 10) to inhibit the cytotoxicity induced by each of the four O/V combinations was evaluated by the MTT assay. Among the O/V combinations tested, OV4 (0.27/0.027 mM O/V ratio) was identified as the most suitable for TTX detection: its cytotoxic effect was significantly counteracted by TTX in a concentration-dependent manner, with a cell viability increase of about 70% at the highest TTX concentration (Figure 1D).

Overall, these results allowed us to set up a final step-by-step optimized protocol to perform the Neuro-2a assay for TTX detection, with the following experimental conditions: 5000 cells/well seeded in complemented RPMI-1640 medium for 24 h before co-exposure to O/V (0.27/0.027 mM) and TTX for 24 h, and subsequent cell viability assessment after 4 h from cell exposure to fresh MTT-containing and O/V-free medium.

### 2.2. Validation of the Neuro-2a Assay

#### 2.2.1. Calibration Curve for TTX

**Figure 2:**
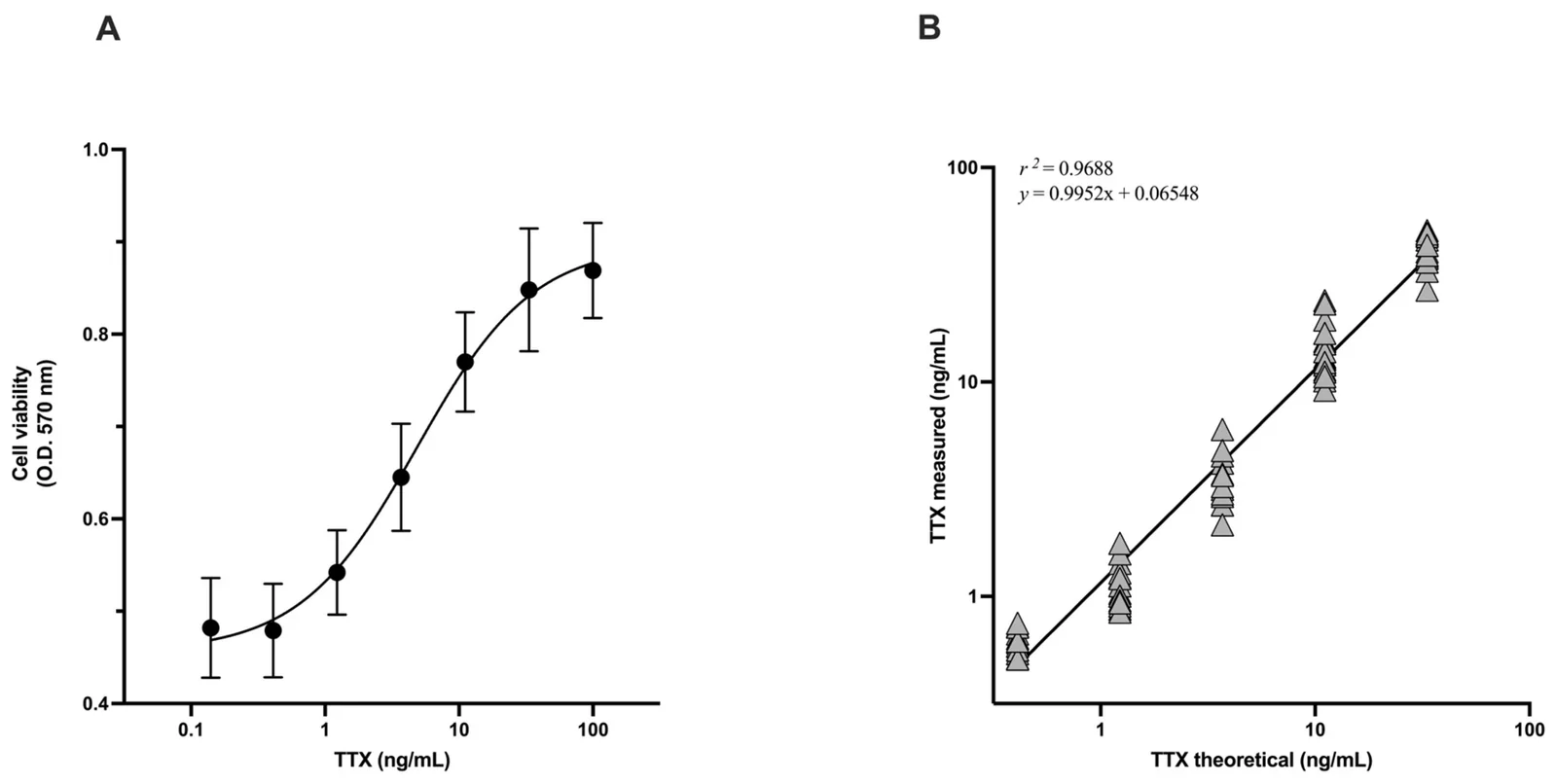
Characterization of the Neuro-2a assay sensitivity for TTX detection. (**A**) Calibration curve of the optimized Neuro-2a assay for TTX detection (each point represents the mean ± SEM; *n* = 10). (**B**) Linear regression analysis performed within the working range of the Neuro-2a assay (0.44–33.3 ng/mL) by plotting the theoretical TTX concentrations against the toxin concentrations measured by the Neuro-2a assay (*n* = 10).

Once an optimized procedure for the Neuro-2a assay was established, seven TTX concentrations were evaluated for their ability to inhibit O/V-induced cytotoxicity, obtaining the calibration curve for TTX represented in Figure 2A (*n* = 10). The working range for TTX detection was 0.44–33.3 ng/mL, with a limit of detection (LOD) and quantitation (LOQ) of 0.132 ng/mL and 0.439 ng/mL, respectively. Analysis of the working range by linear regression, plotting TTX theoretical concentration versus the toxin concentrations measured by the Neuro-2a assay, revealed a good correlation coefficient (*r*^2^ = 0.9737; *n* = 10), indicating good linearity (Figure 2B). A mean *Bias* value (%) of 7.1% (range: −2.4–15.2%) indicated an acceptable accuracy of the measurement.

**Figure 3:**
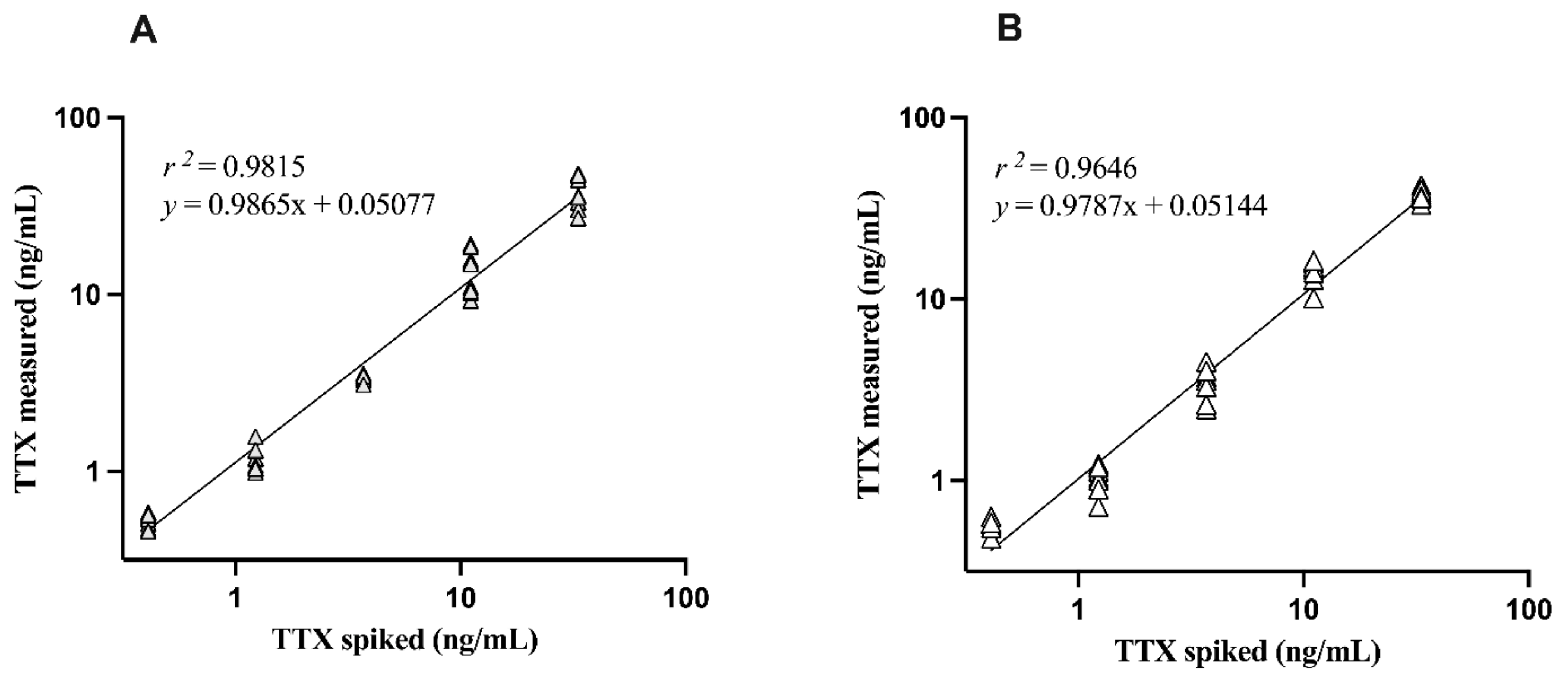
Repeatability of the Neuro-2a assay for TTX detection: linear regression analysis was performed within the working range of the Neuro-2a assay (0.44–33.3 ng/mL) by plotting the theoretical TTX concentrations against toxin concentrations measured by the Neuro-2a assay. (**A**) Intra-day repeatability was evaluated by carrying out the assay within one day by the same operator (*n* = 10). (**B**) Inter-day repeatability was evaluated by carrying out the assay over a 6-month period by different operators (*n* = 10).

The intra-day repeatability, evaluated performing the assay by the same operator (*n* = 10) within the same day (Figure 3A), and inter-day repeatability, evaluated performing the assay by different operators (*n* = 10) within a period of six months (Figure 3B), were determined by the linear regression analysis within the working range of the assay (0.44–33.3 ng/mL). The obtained results show good linearity both for intra-day (*r*^2^ = 0.9815) and inter-day (*r*^2^ = 0.9646) repeatability, supported by the good repeatability coefficients (RSDr = 15 and 11%, respectively).

#### 2.2.2. Cross-Reactivity with Toxins Sharing the Same TTX Mechanism of Action

**Figure 4:**
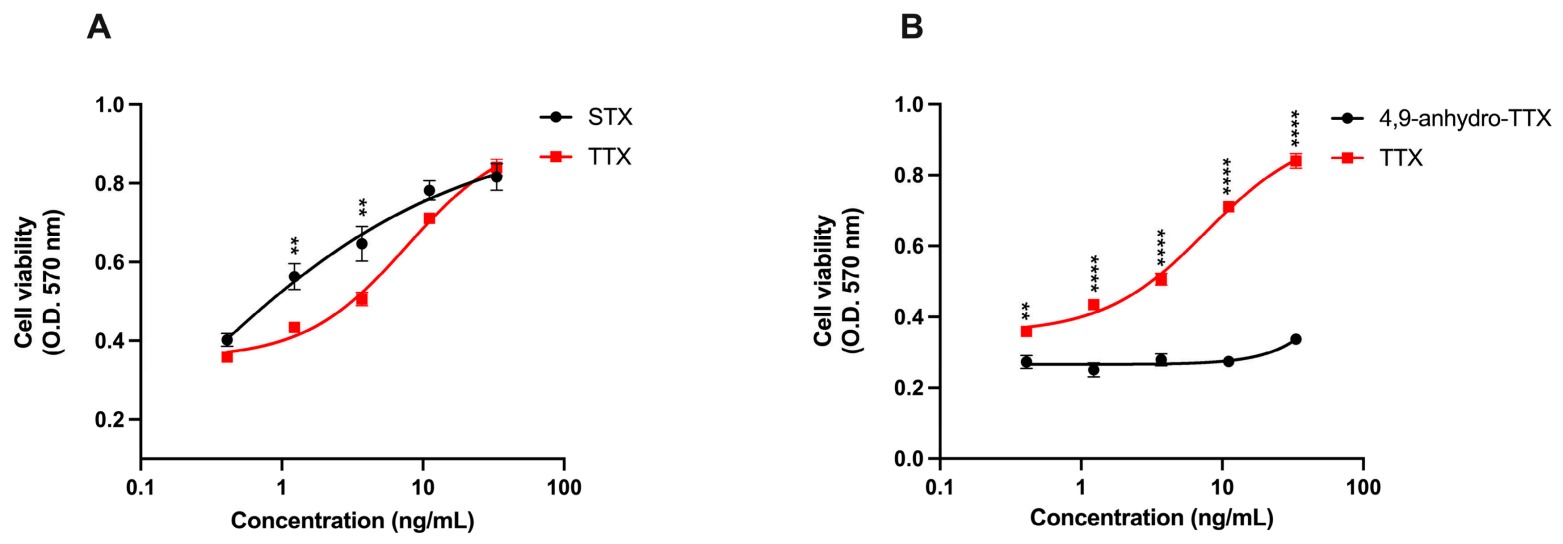
Detection of (**A**) STX and (**B**) 4,9-anhydro-TTX by the optimized Neuro-2a assay in comparison to TTX (0.44–33.3 ng/mL). Each point represents the mean ± SEM of 3 different experiments. Statistical differences vs. TTX: **, *p* < 0.01; ****, *p* < 0.0001 (two-way ANOVA and Bonferroni post test).

The Neuro-2a assay was then evaluated for its ability to detect other marine toxins sharing the same mechanism of action as TTX, the TTX analogue 4,9-anhydro-TTX and saxitoxin (STX). Within the working range of the calibration curve for TTX (0.44–33.3 ng/mL), none of these influenced Neuro-2a cell viability (Figure S1). Thus, 4,9-anhydro-TTX and STX were submitted to the optimized Neuro-2a assay, which detected them with distinct response profiles. In particular, the assay detected STX in a way comparable to that of TTX even if, at concentrations of 1.23 and 3.7 ng/mL, the inhibitory effect of STX on O/V-induced cytotoxicity was significantly higher than that of TTX. At these concentrations, the higher sensitivity of the Neuro-2a assay towards STX may be related to its higher affinity for VGSCs and to a more stable toxin-channel interaction, likely resulting from structural and electrostatic differences between STX and TTX. In fact, as compared with TTX, STX contains an additional guanidinium moiety enabling interaction with an extra carboxylate in domain IV of VGSCs. Consequently, STX exerts a higher inhibitory effect on sodium influx in Neuro-2a cells, as compared with TTX (Figure 4A) [4,45]. On the other hand, the Neuro-2a assay showed very limited effectiveness in detecting 4,9-anhydro-TTX, as demonstrated by its low ability to inhibit the O/V-induced cytotoxicity (Figure 4B). This finding is in agreement with the markedly lower potency of 4,9-anhydro-TTX in comparison to TTX in blocking VGSCs, ascribed to the absence of two hydroxyl groups at positions 4 and 9 [53,54].

#### 2.2.3. Cross-Reactivity with Other Marine Toxins

Cross-reactivity of the Neuro-2a assay was assessed analyzing other marine toxins structurally not related to TTX that may co-occur in edible mussels, such as palytoxin (PLTX), azaspiracid-1 (AZA-1), okadaic acid (OA), dinophysiotoxin (DTX)-1, DTX-2, ciguatoxin (CTX)-1C and CTX-3B, within the working range of the assay (0.44–33.3 ng/mL). The graph reported in Figure S2, illustrating the cytotoxicity of each toxin towards Neuro-2a cells, shows that only AZA-1, CTX-1C and CTX-3B are non-cytotoxic towards these cells. Hence, only these toxins were submitted to the optimized Neuro-2a assay to verify their ability to counteract O/V induced cytotoxicity. As expected, these toxins were not detected by the assay, as they are unable to inhibit the Na^+^-dependent cytotoxicity induced by O/V (Figure S3). In fact, AZA-1 does not act on VGSCs [55], whereas CTX-1C and CTX-3B are activators of VGSCs and, under the adopted experimental conditions, they are unable to counteract O/V-induced cytotoxicity in Neuro-2a cells mediated by sodium ion influx [56]. In the case of CTXs, which are VGSC-activating toxins, an increase in O/V-induced cytotoxicity would be expected. However, this effect was not observed in our study. This can be explained by the O/V concentrations used (0.27/0.027 mM), which were specifically optimized for TTX detection and are considerably higher than those typically employed for CTX detection (approximately 100/10 μM). At these high O/V concentrations, the marked cytotoxicity induced by O/V alone likely masks the additional cytotoxic effect of CTXs, thereby preventing the observation of a further increase in cytotoxicity [51,57,58,59]. In addition, given the hydrophilic nature of TTXs and the lipophilic nature of CTXs, the extraction procedure employed for mussel analysis (see Section 3.6) is expected to preferentially extract TTXs from mussels rather than lipophilic CTXs, thereby reducing the likelihood of CTX-related interference in the Neuro-2a assay.

#### 2.2.4. Matrix Effect

The assay’s suitability to quantify TTX in mussels was investigated. Initially, matrix effect was evaluated to assess whether constituents of mussel extracts could interfere with the Neuro-2a assay. Although the Neuro-2a assay has been applied to different seafood matrices, such as puffer fish and bivalve mollusks, matrix effects have not been previously evaluated in these studies and, consequently, TTX quantitation in mussels by this assay still remains questionable [22,25,48].

**Figure 5:**
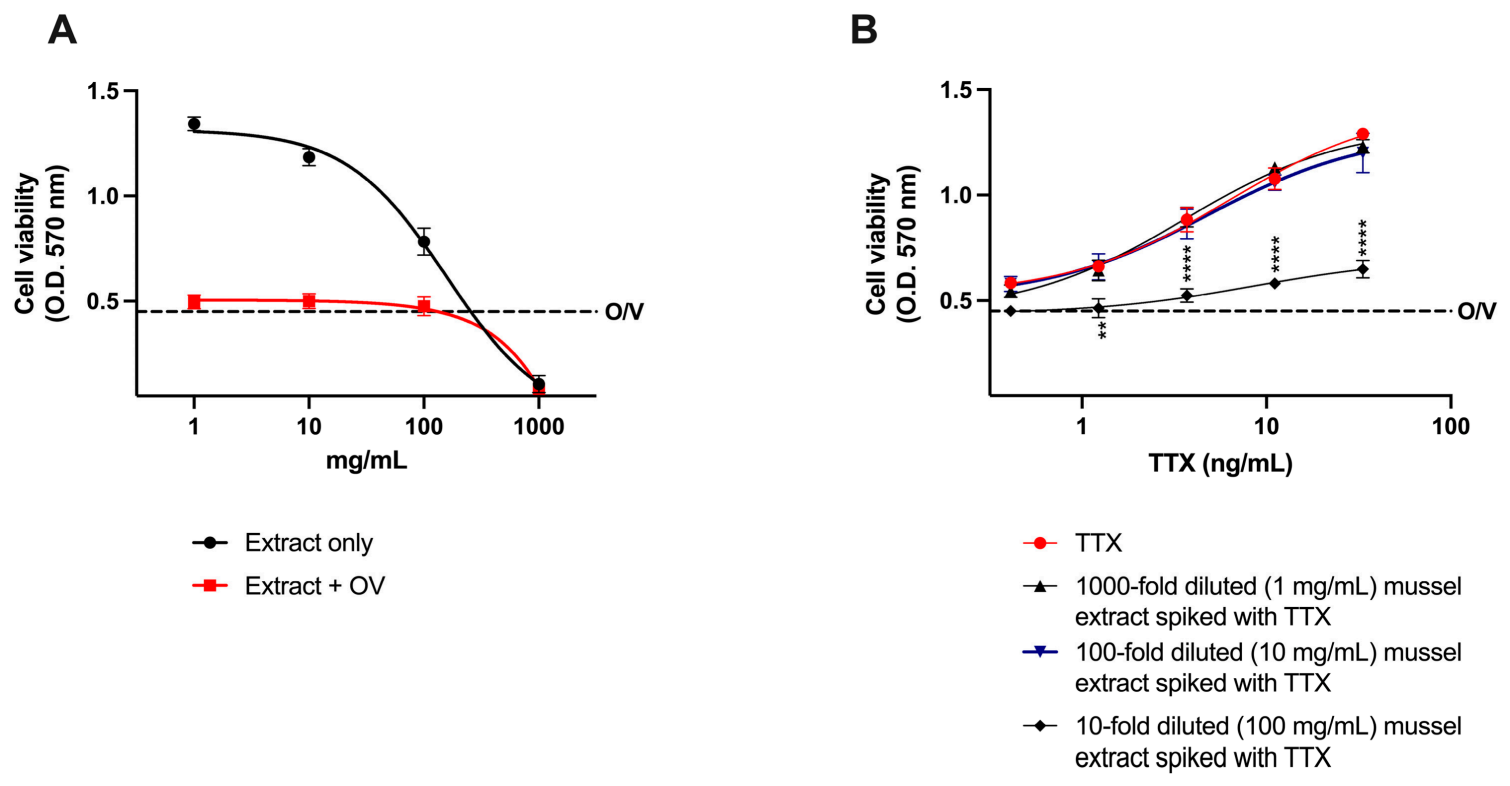
Matrix effect of mussel extract on the Neuro-2a assay. (**A**) Viability of Neuro-2a cells exposed to different dilutions of a mussel extract (1:1000 to 1:1, equivalent to 1–1000 mg mussel meat/mL, respectively) for 24 without (black curve) or with O/V (red curve); the dashed black line represents the cytotoxic effect induced by O/V alone (positive control). (**B**) Viability of Neuro-2a cells exposed to TTX without or with mussel extract at different dilutions (1:1000 to 1:10, equivalent to 1 to 100 mg mussels’ meat/mL, respectively) and O/V (0.27/0.027 mM). Results are shown as optical density values (O.D.) at 570 nm. Each point represents the mean ± SEM of 3 different experiments. Statistical differences vs. TTX: **, *p* < 0.01; ****, *p* < 0.0001 (two-way ANOVA and Bonferroni post test).

Firstly, different extracts from TTX- and STX-free mussels were evaluated for their cytotoxicity toward Neuro-2a cells after 1:1, 1:10, 1:100, 1:1000 (*v*/*v*) dilution (equivalent to 1000, 100, 10 and 1 mg mussel meat/mL, respectively) and for their ability to interfere with O/V (0.27/0.027 mM)-induced cytotoxicity. As shown in Figure 5A (black curve), only 1:100 and 1:1000 extract dilutions (equivalent to 10 and 1 mg mussel meat/mL, respectively) were not cytotoxic for Neuro-2a cells. Moreover, assessment of the impact of diluted mussel extracts on O/V-induced cytotoxicity showed that 1:10 dilution (equivalent to 100 mg mussel meat/mL) was the minimum dilution at which no interference with O/V-induced cytotoxicity was observed (Figure 5A, red curve). Thus, extracts at 1:10, 1:100 and 1:1000 dilutions were subsequently spiked with known amounts of TTX (final concentrations ranging from 0.44 ng/mL to 33.3 ng/mL) and analyzed by the Neuro-2a assay in comparison to matrix-free TTX solutions, at the same toxin concentrations. No matrix-related interferences were observed starting from a 1:100 dilution (equivalent to 10 mg mussel meat/mL). Hence, this dilution was chosen as the lowest dilution of mussel extract not affecting TTX detection by the optimized Neuro-2a assay (Figure 5B).

**Figure 6:**
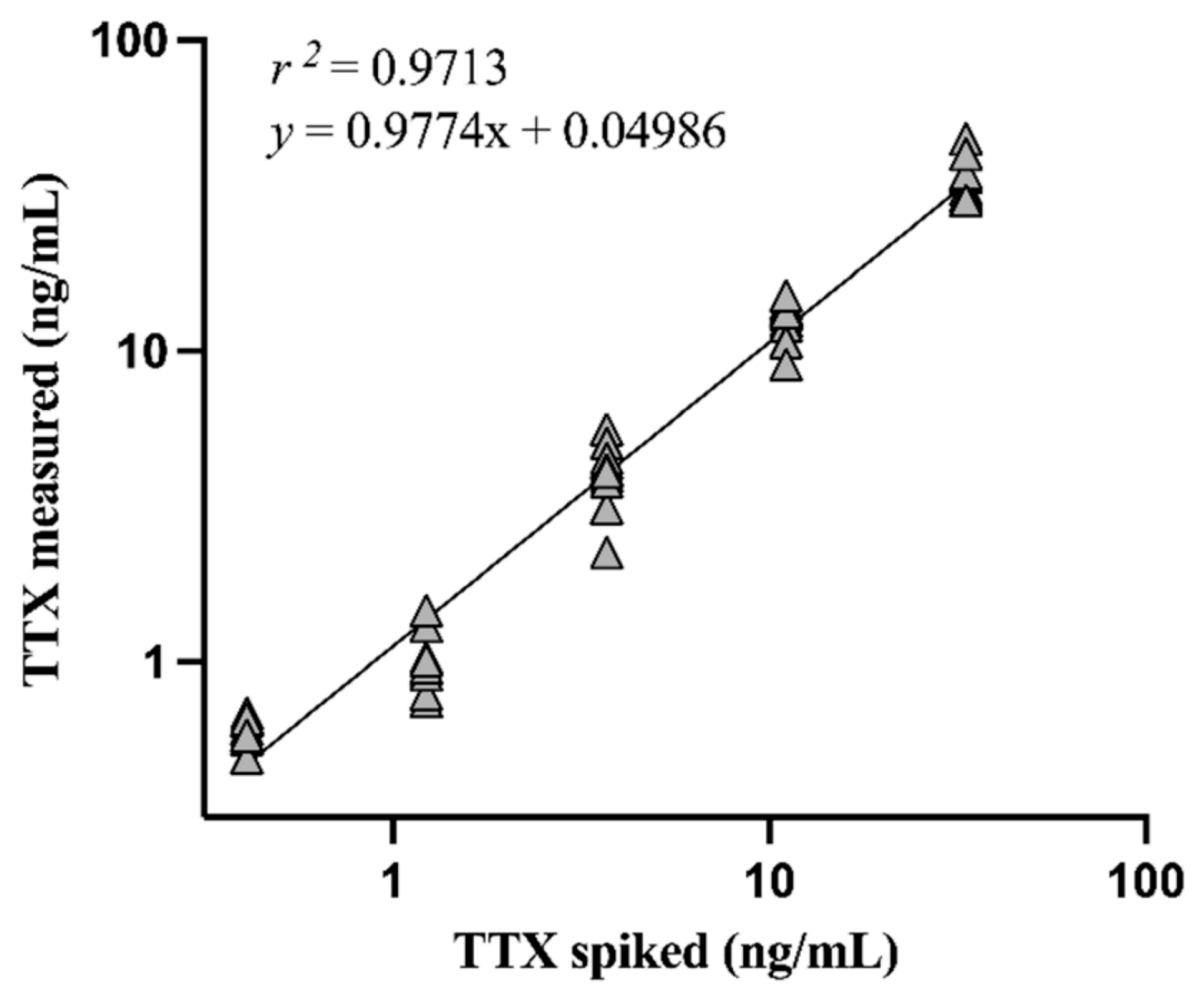
Linear regression analysis of the Neuro-2a assay performed in 100-fold diluted mussel extract spiked with known concentrations of TTX within the working range (0.44–33.3 ng/mL). The theoretical TTX concentrations spiked in the extract are plotted against the toxin concentrations measured by the Neuro-2a assay (*n* = 10).

Subsequently, the performance of the assay to quantify TTX in 100-fold diluted mussel extract was characterized following the Eurachem guidelines [60]. Figure 6 shows the linear regression analysis obtained by comparing the theoretical TTX concentrations in the spiked 100-fold diluted mussel extract to those measured by the Neuro-2a assay. The linear regression analysis yielded a good correlation coefficient (*r*^2^ = 0.9713), demonstrating good linearity and reliability. An acceptable mean Bias of 10.7% was calculated, with Bias at single concentrations ranging between −18.2% and 44.3%. Since a high relative Bias (44.3%) was recorded only at the lowest TTX concentration (0.44 ng/mL), the assay can be suitable within its practical working range. In fact, although the TTX LOD in diluted mussel extract was 0.162 ng/mL, the LOQ was slightly higher than 0.44 ng/mL and equal to 0.540 ng/mL. These values correspond to an LOD of 0.162 µg/kg and to a LOQ of 0.540 µg/kg in diluted mussels’ meat, which are very close to the LOD and LOQ for TTX in matrix-free solution. These results corroborate that the optimized Neuro-2a assay does not suffer from a significant matrix effect when mussels’ extracts are 100-fold diluted. Considering the 1:100 dilution, LOD and LOQ for TTX in mussels correspond to 16.2 and 54 µg/kg meat, respectively, which are lower and slightly higher than the maximum TTX limit recommended by EFSA in shellfish (44 µg TTXs/kg meat) [3]. These results, summarized in Table 1 along with the other validation parameters, suggest that the Neuro-2a assay is sensitive enough to detect TTX at levels comparable to the EFSA-recommended limit, albeit with a non-optimal accuracy for the toxin quantitation at the lower concentration range.

A direct comparison of these LOQ and LOD values with the corresponding values of other functional bioassays is not currently possible, since this Neuro-2a assay appears to be the only functional method that has been characterized for its performance in measuring TTX in mussels. On the other hand, the maleimide-based enzyme-linked immunosorbent assay (mELISA) for TTX detection is the only alternative biological method characterized in terms of matrix effects, with a LOQ reported by the authors equal to 30 µg TTX equivalents/kg mussels [61]. However, despite its apparently higher sensitivity, mELISA is a structural assay that does not provide an estimation of the potential toxicity of a mussel sample, which could contain multiple toxin analogs with different toxic potencies. So, functional assays such as Neuro-2a offer a significant advantage, being directly related to the toxic potential of the sample under analysis.

#### 2.2.5. Quantitation of TTX by Neuro-2a Assay in Field-Collected Mussels

The extracts obtained from 25 bivalve (*M. galloprovincialis*) samples collected along the Italian coasts of Friuli Venezia Giulia and Veneto (North Adriatic Sea) were submitted to the Neuro-2a assay for TTX quantitation in comparison with LC-MS/MS. LC-MS/MS analysis revealed the exclusive presence of TTX in some samples, with no detectable levels of TTX analogues, STX, or STX analogues. Representative chromatograms (one from a TTX-positive and one from a TTX-negative sample) are reported in Supplementary Figure S4. Since the functional Neuro-2a assay could also detect TTX analogues and/or other compounds acting as inhibitors of VGSCs, toxin concentrations measured by the bioassay are expressed as µg TTX equivalents/kg mussel meat, as they were calculated by a calibration curve generated using TTX as the reference toxin. Thus, they represent the amount of TTX that would produce an equivalent functional response under the same assay conditions. Both the Neuro-2a assay and LC-MS/MS analysis revealed the absence of detectable TTXs in 6 of the 25 samples analyzed, whereas TTX levels in 11 samples analyzed by LC-MS/MS were below the LOQ of the Neuro-2a assay, falling within the range of 11–49 µg/kg. In the other 8 samples, TTX concentrations measured by the Neuro-2a assay ranged between 67.8 and 176.6 µg TTX equivalents/kg mussel meat and were comparable to TTX concentrations recorded by LC-MS/MS (58–196 µg/kg mussel meat; Table 2). The parametric Pearson’s correlation analysis of these data showed an optimal positive correlation between the results obtained with the Neuro-2a assay and LC-MS/MS (*r* = 0.9875, *p* ≤ 0.0001). Thus, the Neuro-2a assay for TTX quantification provided results that closely mirror those recorded by the reference analytical method.

Overall, these results demonstrate that the functional Neuro-2a assay allows the detection of TTX-equivalent VGSC-blocking toxins, supporting its suitability as a reliable screening tool to assess the potential presence of TTX and its analogs in mussels, complementary to the instrumental LC-MS/MS method.

## 3. Materials and Methods

### 3.1. Chemicals

Tetrodotoxin (TTX), saxitoxin (STX), dinophysistoxin-1 (DTX-1), dinophysistoxin-2 (DTX-2) and okadaic acid (OA) were purchased from the National Research Council (Ottawa, ON, Canada). Palytoxin (PLTX), ciguatoxin-3C (CTX-3C), and ciguatoxin-1B (CTX-1B) were purchased from Wako Pure Chemicals Industries Ltd. (Osaka, Japan), while 4,9-anhydro-TTX was purchased from Bio-Techne (Milan, Italy). The Neuro-2a cell line was purchased from ATCC (Manassas, VA, USA). All the reagents of analytical grade were purchased from Sigma-Aldrich (Milan, Italy), unless otherwise specified.

### 3.2. Cell Culture

Neuro-2a cells were cultured in RPMI-1640 supplemented with 10% fetal bovine serum (FBS), 1.0 × 10^−2^ M L-glutamine, 1.0 × 10^−4^ g/mL penicillin, 1.0 × 10^−4^ g/mL streptomycin and 1% of 100 mM sodium pyruvate at 37 °C under a humidified 95% air/5% CO_2_ atmosphere. Cell passages were performed two times per week up to approximately 80–90% confluence.

### 3.3. Experimental Design

Considering the protocols for TTX detection by Neuro-2a assay reported in the literature, the functional assay was optimized for TTX detection considering the following non-standardized experimental parameters: (*i*) density of seeded cells; (*ii*) type of culture medium; (*iii*) MTT assay protocol and (*iv*) optimal O/V concentration ratio [22,48,49,50,51]. These parameters were optimized taking into account the main experimental steps of the original assay set up for STX detection [44], as follows: (*i*) cells culture in in 96-well plates for 24 h (cell densities ranging from 1000 to 50,000 cells/well in RPMI-1640 or MEM were considered); (*ii*) cell exposure to O/V and TTX for 24 h (four O/V ratios were used: O/V 1 = 0.125/0.2 mM; O/V 2 = 0.35/0.35 mM; O/V 3 = 0.3/0.03 mM; O/V 4 = 0.27/0.027 mM); (*iii*) evaluation of cell viability by the 3-(4,5-dimethyl-2-thiazol)-2,5-diphenyl-2H-tetrazolium bromide (MTT) assay, considering two procedures: (*1*) direct addition of MTT to the culture medium after 20 h exposure to O/V and further cell incubation for 4 h; (*2*) after 24 h exposure to O/V, culture medium replacement with fresh MTT-containing medium and cell incubation for additional 4 h). The optimized assay was subsequently characterized according to the international principles outlined in the Eurachem Guidelines (Eurachem Guides, 2025) [60].

### 3.4. Optimized Protocol for the Neuro-2a Assay

By evaluating the influence of the aforementioned parameters on the assay performance, the established conditions for the optimized assay are described below. Neuro-2a cells were seeded into 96-well plates at a density of 5 × 10^3^ cells/well in 180 μL of complete RPMI-1640. After 24 h of culture, the calibration curve for TTX detection was obtained by adding 20 μL to each well of cell medium containing TTX standard solution (TTX final concentrations: 0.13–100 ng/mL, dilution factor 3) and O/V, and incubating the cells for an additional 24 h. A ratio of O/V concentrations inducing approximately 80% cell mortality (0.27 mM/0.027 mM, respectively) was used to assess TTX’s ability to counteract cell death. As a positive control, cells were exposed to O/V (0.27 mM/0.027 mM) alone. After treatment, cell viability was assessed by the MTT assay. Briefly, the culture medium was removed and replaced with 100 μL of fresh medium containing 0.5 mg/mL MTT. After 4 h incubation at 37 °C, insoluble formazan crystals were solubilized with dimethylsulfoxide (DMSO; 100 μL/well) and the absorbance was measured at 570 nm, using the automated Microplate Reader FLUOstar^®^ Omega version 5.70 (BMG LABTECH; Ortenberg, Germany). Results are reported as optical density (O.D.) values at 570 nm. Each determination was performed in triplicate.

### 3.5. Cross-Reactivity

The assay cross-reactivity was evaluated by analyzing toxins sharing the same mechanism of action as TTX and other marine toxins structurally not related to TTX that may co-occur in edible mussels. Saxitoxin (STX), 4,9-anhydro-TTX, palytoxin (PLTX), azaspiracid (AZA-1), okadaic acid (OA), dinophysiotoxin-1 (DTX-1), dinophysiotoxin-2 (DTX-2) and ciguatoxin (CTX-1C and CTX-3B) were tested individually by the Neuro-2a assay at concentrations within the working range of the assay for TTX detection (0.44–33.3 ng/mL).

### 3.6. Evaluation of Mussel Matrix Effect

To assess the Neuro-2a suitability to quantify TTX in mussels, different extracts from TTX- and STX-free mussel (*Mytilus galloprovincialis*) edible parts, previously analyzed by LC-MS/MS, were prepared according to the Standard Operating Procedure (SOP) for determination of TTX provided by the European Union Reference Laboratory for Marine Biotoxins [62], spiked with known amounts of TTX, and submitted to the Neuro-2a assay. Briefly, 5 g of mussel meat homogenate was extracted by vortexing with 5 mL of 1% *V*/*V* acetic acid for 90 s, and the homogenate suspension was then heated in a boiling water bath for 5 min. After cooling to room temperature, the sample was vortexed for 90 s and centrifuged at 4000× *g* for 10 min. One mL of the supernatant was mixed with 5 μL of 25% *v*/*v* ammonia and centrifuged at 1000× *g* for 1 min. The extract was cleaned up with Graphitised Carbon SPE (solid phase extraction), conditioning a Supelclean ENVI-Carb cartridge (Supelco; Sigma-Aldrich, Sintra, Portugal) with 3 mL of 20% *v*/*v* acetonitrile and 1% *v*/*v* acetic acid, followed by 3 mL of 0.025% *v*/*v* NH_3_. A 400 µL aliquot of the sample extract was loaded onto the cartridge, which was washed with 700 µL Milli-Q water, and then eluted with 2 mL of 20% *V*/*V* acetonitrile and 1% *V*/*V* acetic acid. The eluted extract was dried using a rotary evaporator and resuspended in culture medium at the concentration of 1 g meat equivalents/mL.

Four dilutions of the so-obtained TTX-free mussel extract (1:1, 1:10, 1:100, and 1:1000, *v*/*v*) were spiked with known amounts of TTX to obtain matrix-matched samples with TTX concentrations ranging from 0.44 to 33.3 ng/mL. These samples were subsequently analyzed by the Neuro-2a assay, as previously described.

### 3.7. Analysis of Field-Collected Mussels by the Neuro-2a Assay

The optimized Neuro-2a assay was evaluated for its suitability to quantify TTXs in field-collected mussels (*M. galloprovincialis*) in comparison to LC-MS/MS. For this purpose, field-collected samples were extracted as described above, and the extracts were diluted 1:100 for their analysis by the Neuro-2a assay, as previously described. Each analysis was carried out in triplicate.

#### 3.7.1. Collection of Field-Contaminated Mussels

**Figure 7:**
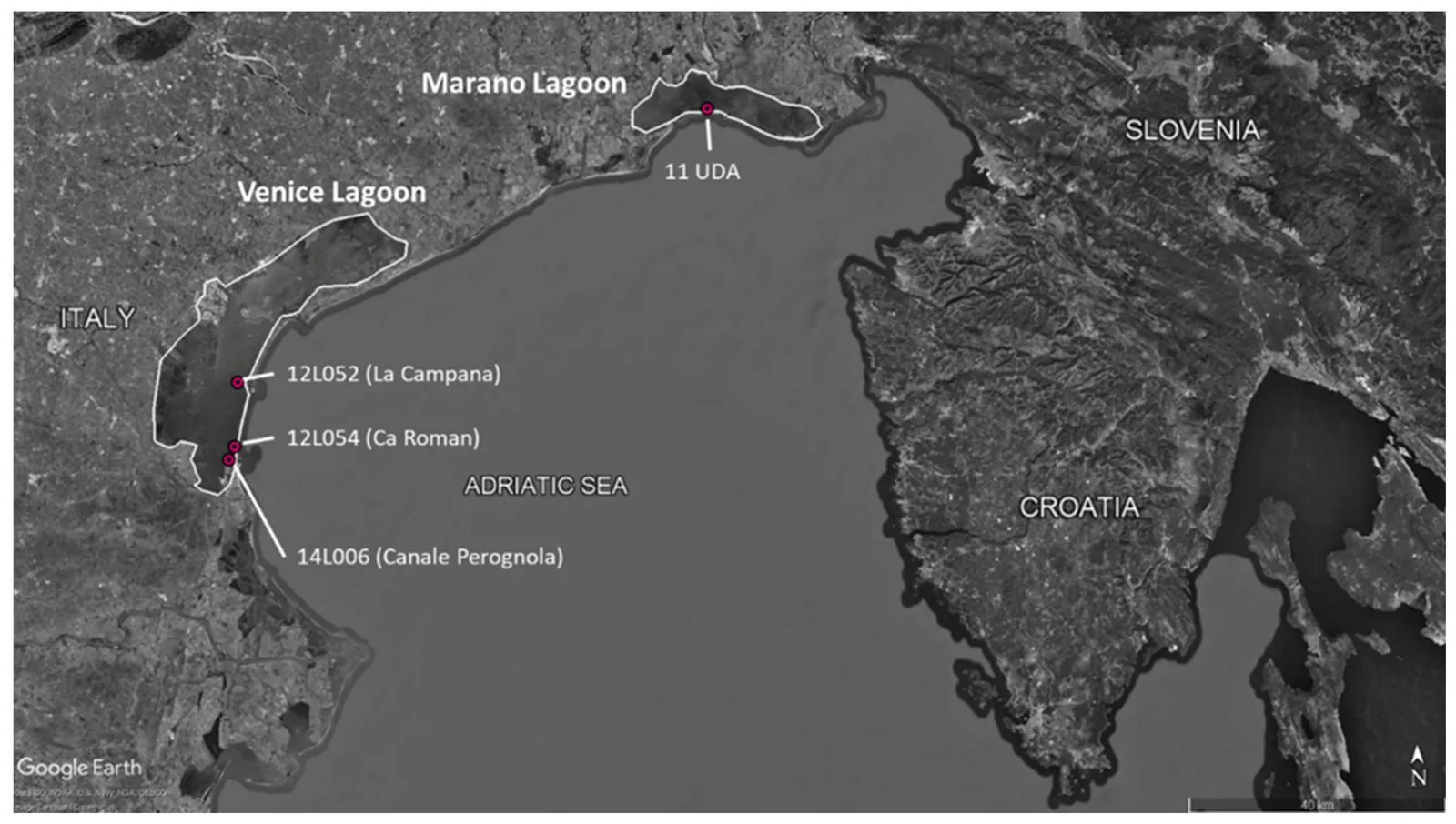
Map showing the sampling points from which field-contaminated mussels were harvested. For clarity, in the map are also highlighted the two lagoons from which the samples were taken: Venice Lagoon and Marano Lagoon. The image was generated by means of Google Earth Pro software 7.3.

Field-contaminated mussels (*M. galloprovincialis*) were collected in lagoon areas from the North Adriatic Sea (Veneto and Friuli-Venezia Giulia regions), from four different sampling points (Figure 7), of which three from the Venice lagoon and one from the Marano lagoon. Samples were collected in specific time periods and with specific frequencies, which were determined based on the data regarding the occurrence of TTXs in shellfish as reported in the work of Antonelli et al. (2023) [63] and Bordin et al. (2021) [26]. Particularly, sampling took place for each of the years 2023, 2024 and 2025 between May and October, with the following frequency: weekly in May and June, fortnightly in July, once a month in August, then weekly in September and fortnightly in October. This was achieved with the support of the local veterinary authorities, which collected the samples from local shellfish farms (only in the Venice lagoon) as part of the local monitoring for microbiological and chemical hazards for live bivalve mollusks destined for human consumption. As for the Marano Lagoon, the area chosen for sampling was already issued by the local veterinary authority to be banned for shellfish farming, so mussels were harvested from wooden poles present within the lagoon to delimit the navigable channels.

At each sampling session, about 2 kg of mussels were taken and stored in ice boxes at 4 °C until their arrival at the laboratory of the Istituto Zooprofilattico Sperimentale delle Venezie (IZSVe) (Adria, Italy), where their external shells were gently brushed and rinsed with distilled water. Then, mussels were shucked with sterile scalpels to collect 100 g of whole flesh for each sample, which was homogenized (Unidrive 1000; Nidec Industrial Automation, Milan, Italy) and stored at −20 °C until identification and quantitation of TTXs.

#### 3.7.2. Analysis of Field-Collected Mussels by LC-MS/MS

Extraction of mussel (*M. galloprovincialis*) meat samples and the clean-up of the obtained extracts were carried out as reported by Antonelli et al. (2023) [63]. Briefly, each homogenized tissue (5 g) was extracted with 5 mL of 1% acetic acid by mixing for 90 s. Then, the suspension was placed firstly for 5 min in a boiling water bath and cooled at room temperature. After vortexing for 90 s, each mixture was centrifuged at 4000× *g* for 10 min. A 1 mL aliquot of each supernatant was transferred into a polypropylene tube and 5 μL of 25% ammonia was added. Each extract was subjected to SPE using ENVI-Carb 250 mg/3 mL cartridges (Sigma-Aldrich, St. Louis, MO, USA) conditioned with 3 mL of 20% acetonitrile and 1% acetic acid, and then with 0.025% ammonia (3 mL). Each extract (400 μL volume) was loaded into the cartridge, washed with 700 μL of water, and then eluted with 2 mL of 20% acetonitrile and 1% acetic acid. A 100 μL aliquot of each eluate was diluted by adding 300 μL of acetonitrile before LC-MS/MS analysis.

The analysis was carried out by injecting 5 µL of the extract into an LC-MS/MS Xevo TQ-XS Triple Quadrupole Mass Spectrometer (Waters Co.; Milford, MA, USA), coupled with UPLC Acquity H.Class (Waters Co.; Milford, MA, USA) in accordance with the European Reference Laboratory for Marine Biotoxins method [62]. TTX certified standard was purchased from CIFGA (Lugo, Spain). All samples were analyzed for the presence of TTX, of its analogues 4-epi-TTX, 5-deoxy-TTX, 11-deoxy-TTX, 4,9-anhydro-TTX, 6,11-dideoxy-TTX and 5,6,11-trideoxy-TTX, according with the applied method.

Simultaneously, the presence of STX and its analogues was assessed using a screening method as reported by Turner et al. (2020) [64]. The method allows the detection of the following toxins: STX, neosaxitoxin (NEO), gonyautoxins (GTX) 1–6, decarbamoyl GTX (dcGTX) 1–4, decarbamoyl STX (dcSTX), decarbamoyl NEO (dcNEO), deoxydecarbamoyl STX (dcSTX), and N-sulfocarbamoyl GTX 1–4 (C1–C4). STX certified standards were purchased from the National Research Council of Canada (Halifax, NS, Canada). The monitored MRM transitions for TTXs and STXs and the LC and MS setting conditions are reported in Supplementary Table S1.

### 3.8. Statistical Analysis

Results of the Neuro-2a assay are presented as mean ± standard error of the mean (SEM) from at least three independent experiments performed in triplicate. Linearity (r^2^) of the calibration curve was calculated by linear regression analysis, using the GraphPad Prism software version 10.0 (GraphPad Prism; GraphPad Software, Inc.; San Diego, CA, USA).

Concentration-effect curves were compared and analyzed by two-way analysis of variance (ANOVA) followed by Bonferroni’s test, and significant differences were considered at *p* < 0.05. The correlation between TTX concentrations determined by the Neuro-2a assay and LC-MS/MS was evaluated using Pearson’s parametric correlation analysis. Correlation was expressed as the Pearson correlation coefficient (*r*), and statistical significance was considered at *p* < 0.05 (GraphPad Prism; GraphPad Software, Inc.; San Diego, CA, USA).

The Neuro-2a assay performance was characterized according to the international principles described by the Eurachem Guides (Eurachem Guides, 2025) [60]. The limit of detection (LOD) and the limit of quantitation (LOQ) were calculated multiplying for 3- and 10-time, respectively, the standard deviation (s’_0_). These values were obtained from the standard deviation of the mean values of 10 blank samples following the formula recommended in the Eurachem Guidelines: s’_0_ $=\frac{s0}{\sqrt{n}}$, where s_0_ is the estimated standard deviation of individual measurement results from replicate determinations at or near zero concentration and *n* is the number of replicates averaged when reporting results where each replicate is obtained following the entire measurement procedure (Eurachem Guides, 2025) [60].

The accuracy was measured as % Bias (*n* = 10), calculated as the % difference between the TTX concentration measured by the assay and the theoretical concentration in the sample divided by the TTX theoretical concentration. Repeatability was expressed as the relative standard deviation of repeatability (RSDr), measured as a % ratio between the standard deviation of independent results and their mean value. Both independent results obtained by the same operator in one day (intra-assay RSDr; *n* = 10) and within a 6-month period by different operators (inter-assay RSDr; *n* = 10) were considered.

## 4. Conclusions

Overall, this study provides an optimized Neuro-2a assay for TTX detection and quantitation in mussels. Given the frequent discrepancies related to different protocols of this functional cell-based assay reported in the literature, the most suitable experimental conditions were established to set up a standardized protocol for optimal assay performance. The intra-laboratory validation according to Eurachem standardized guidelines showed good linearity, sensitivity, and repeatability of the optimized Neuro-2a assay. As expected, it can also detect toxins sharing the same mode of action as TTX (i.e., STX, 4,9-anhydro-TTX), even though with different potencies that appear related to their potencies in blocking the VGSCs and, consequently, to their toxic potential. On the contrary, other marine toxins acting by different mechanisms are not detected by the assay. The potential presence of other toxins cytotoxic to Neuro-2a cells can be assessed by evaluating the cytotoxicity of the mussel samples in parallel, in the absence of O/V.

The analysis of mussel extracts demonstrated that the assay can also be adopted to detect and quantify TTX in mussels after a 100-fold dilution of their extract. At this condition, the assay LOD and LOQ were equal to 0.162 ng/mL and 0.540 ng/mL, respectively, corresponding to 16.2 and 54.0 µg TTX equivalents/kg mussel meat. These values are, respectively, lower and slightly higher than the maximum limit of TTXs recommended by EFSA in mussels (44 µg TTXs/kg meat). Although a high Bias was recorded when analyzing the lowest TTX concentration in the presence of mussel extract (0.44 ng/mL), this did not appear to adversely affect the overall performance of the assay, as this toxin concentration falls at the lower limit of the validated working range, below the LOQ of 0.540 ng/mL. This was also supported by the strong correlation between the TTX concentrations measured by the Neuro-2a assay and those determined by LC-MS/MS (Pearson’s correlation analysis: *r* = 0.9875, *p* ≤ 0.0001).

Overall, even though an inter-laboratory validation should be carried out to assess its reproducibility, the optimized Neuro-2a assay represents a promising screening tool for the assessment of mussel safety related to TTXs, as a complementary analysis to LC-MS/MS, currently proposed as the reference method, to estimate the toxic potential of the analyzed samples.

## Figures and Tables

**Table 1:** Summary of the validation parameters of the optimized Neuro-2a assay for TTX determination.

Parameter	Condition	Result
Working range	TTX in solution	0.44–33.3 ng/mL
	TTX in mussel meat	44–3330 µg/kg
LOD	TTX in solution	0.132 ng/mL
	TTX in mussel meat	16.2 µg/kg
LOQ	TTX in solution	0.439 ng/mL
	TTX in mussel meat	54 µg/kg
Linearity	TTX in solution	*r*^2^ = 0.9737
	TTX in mussel meat	*r*^2^ = 0.9713
Accuracy	TTX in solution	Bias: 7.1% (range: −2.4–15.2%)
	TTX in mussel meat	Bias: 10.7% (range: −18.2–44.3%)

**Table 2:** TTX concentration in mussel samples measured by the Neuro-2a assay and LC-MS/MS.

Sample	Sampling Site	Sampling Month/Year	TTXs Concentration	
			LC-MS/MS (µg TTX/kg)	Neuro-2a Assay (µg TTX Equivalents/kg)
1	Laguna di Marano (11UDA)	05/2023	113	103
2	Laguna di Marano (11UDA)	05/2023	196	177
3	Pellestrina-Mitilla	06/2023	58	67
4	Ca’ Roman (12L054)	05/2025	156	133
5	Ca’ Roman (12L054)	05/2024	96	92
6	Ca’ Roman (12L054)	05/2025	131	122
7	Ca’ Roman (12L054)	05/2024	72	79
8	Pellestrina-Mitilla	05/2023	75	84
9	La Campana (12L052)	06/2023	49	<LOQ ^2^
10	Canale Perognola (14L006)	06/2023	35	<LOQ ^2^
11	La Campana (12L052)	05/2023	28	<LOQ ^2^
12	La Campana (12L052)	05/2023	32	<LOQ ^2^
13	La Campana (12L052)	05/2023	39	<LOQ ^2^
14	Canale Perognola (14L006)	05/2023	45	<LOQ ^2^
15	La Campana (12L052)	06/2023	18	<LOQ ^2^
16	Pellestrina-Mitilla	06/2023	23	<LOQ ^2^
17	Pellestrina-Mitilla	06/2023	14	<LOQ ^2^
18	La Campana (12L052)	06/2023	12	<LOQ ^2^
19	Ca’ Roman (12L054)	06/2025	11	<LOQ ^2^
20	La Campana (12L052)	05/2023	<LOQ ^1^	<LOQ ^2^
21	Pellestrina-Mitilla	07/2023	<LOQ ^1^	<LOQ ^2^
22	Laguna di Marano (11UDA)	06/2024	<LOQ ^1^	<LOQ ^2^
23	La Campana (12L052)	08/2024	<LOQ ^1^	<LOQ ^2^
24	Ca’ Roman (12L054)	10/2025	<LOQ ^1^	<LOQ ^2^
25	La Campana (12L052)	09/2025	<LOQ ^1^	<LOQ ^2^

^1^ LOQ of LC/MS-MS: 10 µg TTX/kg; ^2^ LOQ of the optimized Neuro-2a assay: 54 µg TTX /kg.

## Data Availability

Dataset available on request from the authors.

## Supplementary Materials

The following supporting information can be downloaded at: https://www.mdpi.com/article/10.3390/md24090314/s1, Figure S1: Cytotoxicity induced by TTX and toxins with the same mechanism of action of TTX (STX and 4,9 anhydro-TTX) on Neuro-2a cells, tested within the working range of Neuro-2a assay for TTX (0.44–33.3 ng/mL) for 24 h; Figure S2: Cytotoxicity induced by toxins structurally unrelated to TTX on Neuro-2a cells, tested within the working range of Neuro-2a assay for TTX (0.44–33.3 ng/mL) for 24 h; Figure S3: Detection of AZA-1, CTX-1C and. CTX-3B by the optimized Neuro-2a assay in comparison to TTX (0.44–33.3 ng/mL); Figure S4: Representative chromatograms from a TTX-positive (A) sand from a TTX-negative (B) sample; Table S1: Main LC-MS/MS operating conditions and instrumental characteristics.

## Abbreviations

The following abbreviations are used in this manuscript:

TTX	Tetrodotoxin
VGSCs	Voltage-gated sodium channels
EU	European Union
CBAs	Cell-based assays
LOD	Limit of detection
LOQ	Limit of quantitation
mELISA	Maleimide-based enzyme-linked immunosorbent assay
O	Ouabain
V	Veratridine
O.D.	Optical density
STX	Saxitoxin
PLTX	Palytoxin
AZA-1	Azaspiracid-1
OA	Okadaic Acid
