# Supplementary material for: Optimization and Intra-Laboratory Validation of the Neuro-2a Assay for Tetrodotoxin Detection in Mussels
Source: Mar Drugs. 2026 Sep 8;24(9):314. doi: 10.3390/md24090314 (PMC13608517; doi:10.3390/md24090314)
Supplement: Supplementary file 1 [file marinedrugs-24-00314-s001.zip › marinedrugs-4504759-supplementary.pdf]

Optimization and intra-laboratory validation of the Neuro-2a assay for tetrodotoxin detection in mussels

Alessandra D'Arelli[1], Silvio Sosa[1], Sonia Dall'Ara[2], Monica Cangini[2], Michela Carlin[1], Antonelli Pietro[3,5], Aurora Dall'Occo[3], Nicolas Scapi[4], Giuseppe Arcangeli[4], Carmen Losasso[3], Aurelia Tubaro[1], Marco Pelin[1]*

[1] University of Trieste, Department of Life Sciences, Via A. Fleming 22, 34127 Trieste, Italy
[2] National Reference Laboratory for Marine Biotoxins, Fondazione Centro Ricerche Marine, V.le A. Vespucci 2, 47042 Cesenatico (FC), Italy
[3] Laboratory of Microbial Ecology and Genomics, Istituto Zooprofilattico Sperimentale delle Venezie, Viale dell'Università 10, 35020 Legnaro, Italy
[4] National Reference Center for fish, mollusc and crustacean diseaseas, Istituto Zooprofilattico Sperimentale delle Venezie, Viale dell'Università 10, 35020 Legnaro, Italy
[5] PhD National Programme in One Health Approaches to Infectious Diseases and Life Science Research, Department of Public Health, Experimental and Forensice Medicine, University of Pavia, Pavia 27100, Italy

*Correspondence: mpelin@units.it

Supplementartry data:

Figure S1. Cytotoxicity induced by TTX and toxins with the same mechanism of action of TTX (STX and 4,9 anhydro-TTX) on Neuro-2a cells, tested within the working range of Neuro-2a assay for TTX (0.42-33.3 ng/mL) for 24 h. Results are shown as % of cell viability with respect to untreated controls and are the mean ± SEM of at least three independent experiments performed in triplicate.

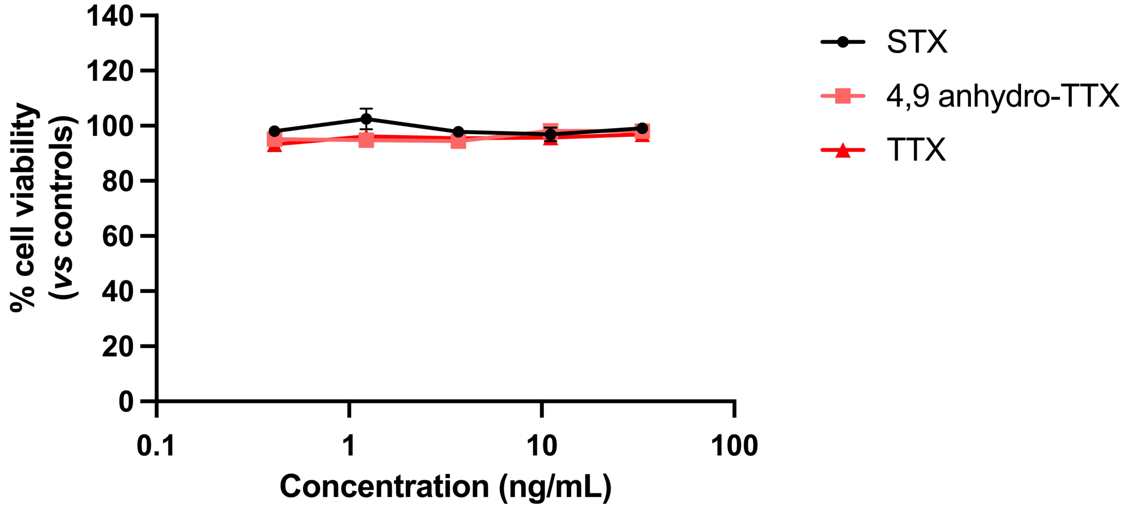

Figure S2. Cytotoxicity induced by toxins structurally unrelated to TTX on Neuro-2a cells, tested within the working range of Neuro-2a assay for TTX (0.42-33.3 ng/mL) for 24 h. Results are shown as % of cell viability with respect to untreated controls and are the mean ± SEM of at least three independent experiments performed in triplicate.

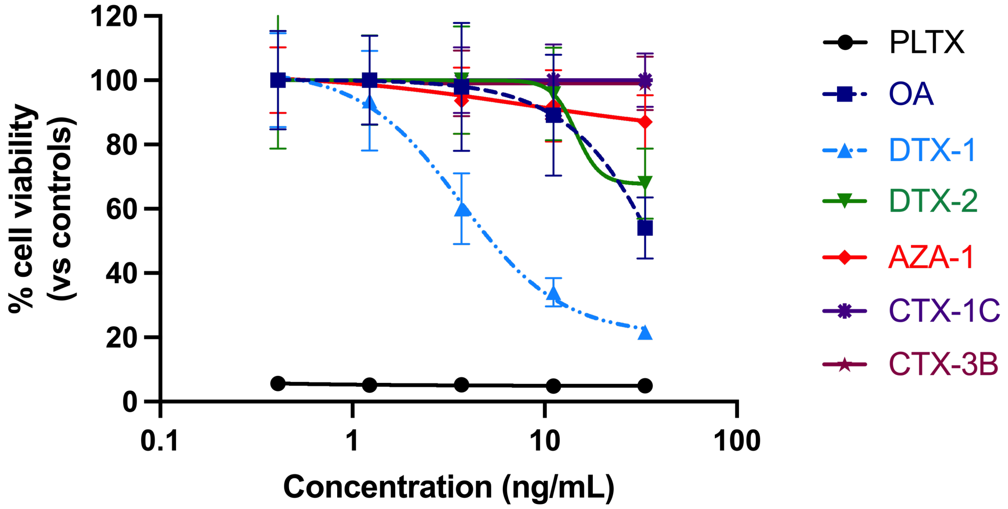

Figure S3. Detection of AZA-1, CTX-1C and. CTX-3B by the optimized Neuro-2a assay in comparison to TTX (0.44-33.3 ng/mL). Each point represents the mean ± SEM of 3 different experiments. Statistical differences *vs* TTX:; ****, *p*<0.0001 (two-way ANOVA and Bonferroni post test).

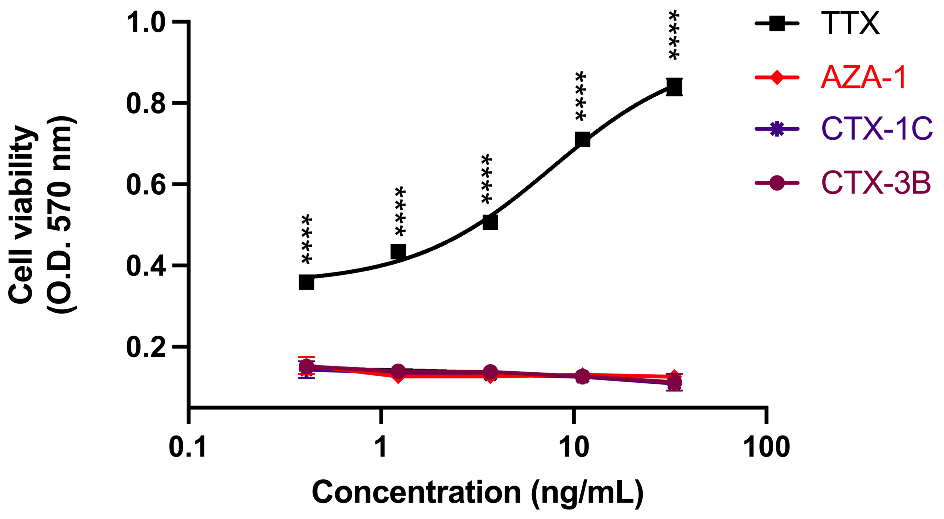

Figure S4. Representative chromatograms from a TTX-positive (A) and from a TTX-negative (B) mussel sample.

A

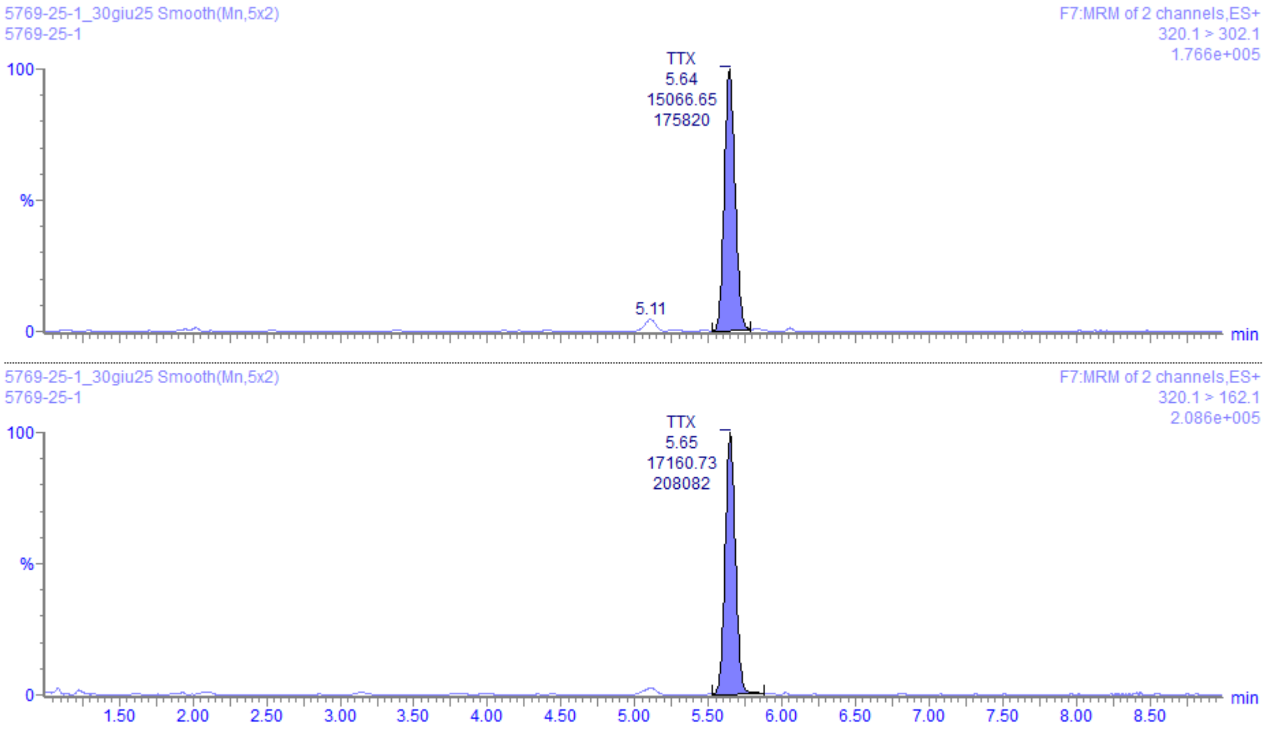

B

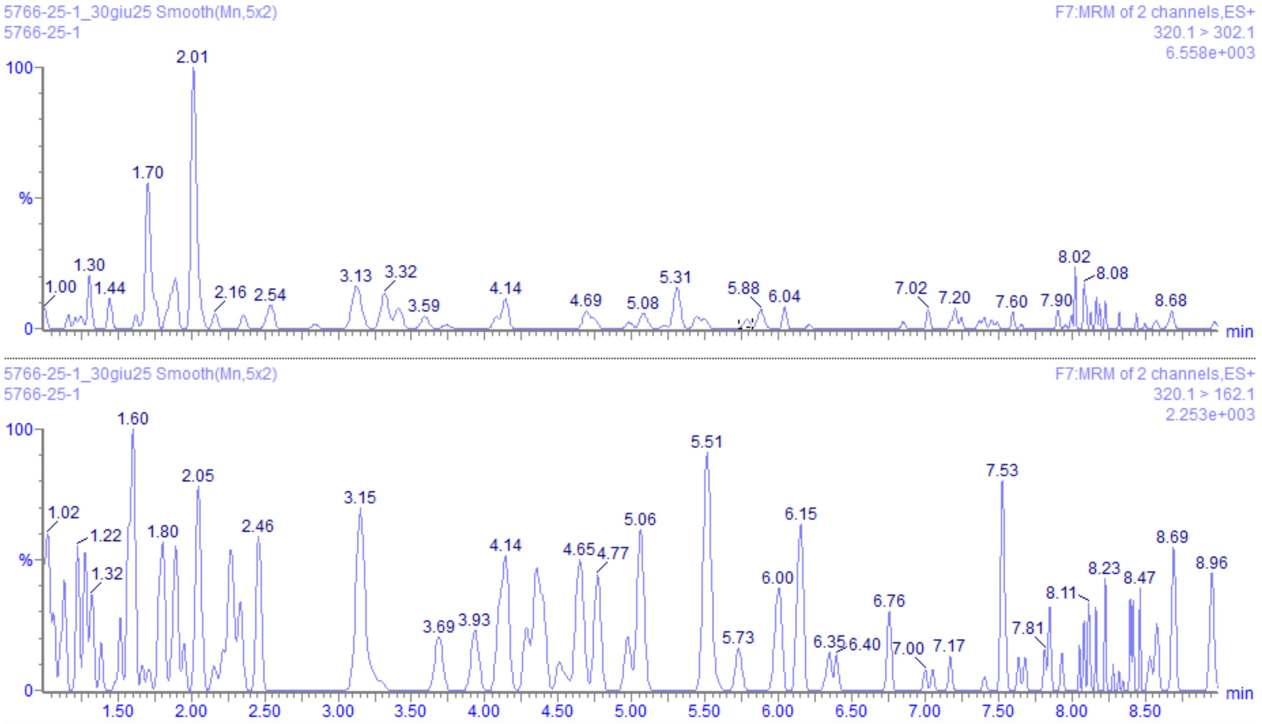

Table S1. Main LC-MS/MS operating conditions and instrumental characteristics.

LC-MS/MS	
BRAND	Waters
MODEL	XEVO TQ XS
Sorgent	ESI SOURCE
Ionization mode	ESI POS/NEG

COLUMN		BRAND
Description	ACQUITY UPLC Glycan BEH Amide 1,7 µm 2,1 µm x 150mm	WATERS
Guard-cartridge	assy acquity guard filter waters pn 289002378 con frit 0,2 micron 2,1 mm	WATERS
Temperature (°C)	60°C	
Max pressure (bar)	900	

Autosampler	
Model	ACQUITY UPLC SAMPLE MGR FTN
Injection Volum	5 µL
Temperature (°C)	10°C

UHPLC mobile phase	
A	500 mL Water +0,100 mL Formic Acid +0,300 mL ammonium hydroxide 25%
B	700 mL acetonitrile+ 300 mL water, +0.1 mL Formic Acid

MS PARAMETERS	+	-
**VOLTAGE**		
Capillary	3	1,8
Cone	20	102,0
Desolvation temeprature	500	500,0
**GAS FLOW**		
Desolvation /L/Hr)	1000	1.000,0
Cone (L/Hr)	150	150,0
Nebulizer (bar)	7	7,0
**ANALYZER**	/	
LM Resolution 1	2,71	2,67
HM Resolution 1	15,0	15,14
Ion Energy 1	0,6	0,60
LM Resolution 2	2,75	2,72
HM Resolution 2	14,82	15,01
Ion Energy 2	0,8	1,00
Collision gas flow (ml/min)	0,15	0,15
Collision	4,0	4,00
**SORGENT**		
Source Temp.	150	150
Ion Guide Offset (1)	3,0	3,0
Ion Guide offset (2)	0,3	0,3
Entrance	30	30
Exit	30	30
GAIN	1,0	1,0

LC METHOD for Analysis				
Time	flow (ml/min)	FMA	FMB	curve
Initial	0,400	2	98	Initial
2	0,400	2	98	6
4	0,400	5	95	6
7,5	0,400	50	50	6
9	0,400	50	50	6
9,3	0,400	2	98	6
9,8	0,400	2	98	6
10,6	0,400	2	98	6
11	0,400	2	98	1
run time	11 min			

TTXs transitions

TTXs-MRM transitions	type	Parent (m/z)	Daughter (m/z)	Cone (V)	Collision (eV)	Polarity
TTX	QUANT	320,1	302,1	40	25	Positive
TTX	QUAL	320,1	162,1	40	38	Positive
epi-TTX	QUANT	320,1	302,1	40	25	Positive
epi-TTX	QUAL	320,1	162,1	40	38	Positive
5,6,11-Trideoxy-TTX	QUANT	272,1	254,1	40	30	Positive
5,6,11-Trideoxy-TTX	QUAL	272,1	162,1	40	30	Positive
6,11-dideoxy-TTX	QUANT	290,1	272,1	40	30	Positive
6,11-dideoxy-TTX	QUAL	290,1	162,1	40	30	Positive
4,9-anhidroTTX	QUANT	302,1	284,1	40	30	Positive
4,9-anhidroTTX	QUAL	302,1	162,1	40	30	Positive
11-deoxy-TTX	QUANT	304,1	286,4	40	30	Positive
11-deoxy-TTX	QUAL	304,1	162,1	40	30	Positive

PSP transitions

Compound Name	type	Parent (m/z)	Daughter (m/z)	Cone (V)	Collision (eV)	Polarity
C1	QUANT	474	121,89	46	30	Negative
C1	QUAL	474	351,1	47	25	Negative
C3	QUANT	490,1	410,1	40	20	Positive
C3	QUAL	412,1	332,1	30	16	Negative
C2	QUANT	396,1	298,13	16	20	Positive
C2	QUAL	474	122	46	30	Negative
C4	QUANT	412,1	314,1	40	20	Positive
C4	QUAL	490,1	392,1	30	20	Negative
GTX2	QUANT	394,0681	351,1001	50	16	Negative
GTX2	QUAL	394,0681	333,1	50	22	Negative
GTX1	QUANT	410,0681	367,1	46	15	Negative
GTX1	QUAL	410,0681	349,1	15	22	Negative
dcGTX2	QUANT	351,0042	164,015	52	30	Negative
dcGTX2	QUAL	351,0042	333,1	40	17	Negative
GTX3	QUANT	394,1	333,1	50	22	Negative
GTX3	QUAL	396,1958	298,1337	2	18	Positive
GTX4	QUANT	410,0681	367	46	15	Negative
GTX4	QUAL	412,1	314,1	30	20	Positive
dcGTX3	QUANT	353,1958	255,1703	2	18	Positive
dcGTX3	QUAL	351,05	333,1	40	18	Negative
GTX5	QUANT	380,13	300,15	6	16	Positive
GTX5	QUAL	378	122	18	25	Negative
GTX6	QUANT	396,1	316,1	30	15	Positive
GTX6	QUAL	394,1	122	40	25	Negative
doSTX	QUANT	241,1	60	40	23	Positive
doSTX	QUAL	241,1	206,1	40	22	Negative
dcSTX	QUANT	257,0041	125,9959	10	19	Positive
dcSTX	QUAL	257,0041	222	10	22	Positive
dcNEO	QUANT	273,0681	125,9978	20	20	Positive
dcNEO	QUAL	273,0681	225,1	20	18	Positive
STX	QUANT	300,2597	204,1	50	23	Positive
STX	QUAL	300,2597	138,1	60	30	Positive
NEO	QUANT	316,1319	126	60	26	Positive
NEO	QUAL	316,1319	220,1	40	23	Positive

QUANT for quantification use
QUAL for confirmation use
